# Aging and Memory as Discrimination: Influences of Encoding Specificity, Cue Overload, and Prior Knowledge

**DOI:** 10.1037/pag0000126

**Published:** 2016-11

**Authors:** Stephen P. Badham, Marie Poirier, Navina Gandhi, Anna Hadjivassiliou, Elizabeth A. Maylor

**Affiliations:** 1 Division of Psychology, Nottingham Trent University; 2 Department of Psychology, City University London; 3 Department of Psychology, University of Warwick

**Keywords:** aging, associative memory, encoding-retrieval match, memory as discrimination, schemas

## Abstract

From the perspective of memory-as-discrimination, whether a cue leads to correct retrieval simultaneously depends on the cue’s relationship to (a) the memory target and (b) the other retrieval candidates. A corollary of the view is that increasing encoding-retrieval match may only help memory if it improves the cue’s capacity to discriminate the target from competitors. Here, age differences in this discrimination process were assessed by manipulating the overlap between cues present at encoding and retrieval orthogonally with cue–target distinctiveness. In Experiment 1, associative memory differences for cue–target sets between young and older adults were minimized through training and retrieval efficiency was assessed through response time. In Experiment 2, age-group differences in associative memory were left to vary and retrieval efficiency was assessed through accuracy. Both experiments showed age-invariance in memory-as-discrimination: cues increasing encoding-retrieval match did not benefit memory unless they also improved discrimination between the target and competitors. Predictions based on the age-related associative deficit were also supported: prior knowledge alleviated age-related associative deficits (Experiment 1), and increasing encoding-retrieval match benefited older more than young adults (Experiment 2). We suggest that the latter occurred because older adults’ associative memory deficits reduced the impact of competing retrieval candidates—hence the age-related benefit was not attributable to encoding-retrieval match per se, but rather it was a joint function of an increased probability of the cue connecting to the target combined with a decrease in competing retrieval candidates.

This article explores how aging affects the discrimination process that is often thought to be at the heart of retrieval from memory. Discrimination here relates to a cue’s capacity to elicit a retrieval target while excluding competing candidates (e.g., Capaldi & Neath, 1995; Craik & Jacoby, 1979; Eysenck, 1979; Hunt, 2003; Nairne, 2002; Surprenant & Neath, 2009).

A long-standing principle of memory is that of encoding specificity. Thomson and Tulving (1970) demonstrated that the most effective retrieval cues in a memory task are those that were processed at encoding. After studying weakly associated cue–target pairs (e.g., train-black), participants were better at cued recall of targets with the original cues compared with extraexperimental cues that were strongly associated to the targets (e.g., white). Based on this study and others, Tulving (1983, p. 224) suggested the following:

The essence of the set of ideas known as encoding specificity lies in the emphasis on the interaction between the stored information and the retrieval information [. . .]. The engram of an event stored in the episodic system, and the retrieval cue, as interpreted or encoded in light of the information in the semantic system, must be compatible for remembering to occur. There are many ways of thinking about the compatibility of the relation [. . .]. But the relation itself is all important in the sense that if it does not exist, recollection of the event will fail.

As mentioned above, many if not most memory researchers would also insist that retrieval is a discrimination process: to be useful, a cue must elicit the to-be-recalled (TBR) information while also allowing the elimination of other potential retrieval targets. In other words, what is central to successful retrieval is the distinctiveness of the cue–target relationship (e.g., Capaldi & Neath, 1995; Craik & Jacoby, 1979; Eysenck, 1979; Hunt, 2003). One of the implications of the memory-as-discrimination view highlighted by some researchers (e.g., Nairne, 2002, 2005; Tulving, 1983) is that factors such as encoding-retrieval match cannot have a causal relationship per se with retrieval. Note that encoding specificity is not synonymous with encoding-retrieval match (see Nairne, 2002; Poirier et al., 2012; Surprenant & Neath, 2009; Tulving, 1983, for more thorough discussions of this distinction). In general, successful memory is viewed by the encoding specificity principle as a joint function of the traces formed during encoding and their interaction with information available at retrieval (Brown & Craik, 2000; Tulving, 1984), whereas encoding-retrieval match is a more general concept that suggests that the probability of retrieval is a monotonically increasing function of the overlap between the cues present at encoding and those present at test. As mentioned above, Nairne (2002, 2005) championed the point that match in itself cannot predict retrieval; if the increase in match involves nondiagnostic information—that is, information related to multiple targets—then it could hurt rather than improve performance. Hence, the memory-as-discrimination view also considers cue overload—and more generally competition in retrieval—where retrieval cues have reduced discriminative efficacy if they are related to multiple targets (Watkins & Watkins, 1975).

## The Current Study

Paradigms designed to test the predictions of the memory-as-discrimination view offer new opportunities to understand age-related memory deficits, which are among the most salient and widely investigated age-related cognitive changes (Zacks, Hasher, & Li, 2000). Recent research by Goh and Lu (2012) and Poirier et al. (2012) experimentally manipulated encoding-retrieval match and cue overload in the same design and provided data consistent with the memory-as-discrimination view. Goh and Lu found that providing extra cue information at retrieval (e.g., ‘a four-footed animal’) did not improve performance when that information had no diagnostic value (e.g., all targets were animals with four feet). Moreover, Poirier et al. found that reinstating a larger proportion of the encoding environment at retrieval could *hinder* memory performance when the extra information had no diagnostic value. For example, in Poirier et al.’s Experiment 4, participants studied triplets of pictures (two cues and a target) where two of the pictures would be used in a later recognition task as cues for the third picture (the target). Memory was tested by presenting one or two of the cues at retrieval (i.e., low or high encoding-retrieval match) and participants selected the corresponding target. The main performance measure was response time (RT). Poirier et al. trained participants so that the accuracy of performance was high; they reasoned that cuing effects could then be attributed more reliably to retrieval than to lapses in encoding. Some of the cues predicted more than one target (i.e., they were overloaded/shared cues) and therefore offered no diagnostic value for distinguishing between those targets when later presented at retrieval. Adding a shared cue to a more diagnostic one actually hindered memory performance, even though adding a shared cue reinstated a larger proportion of the encoding context. Basically, Poirier et al. manipulated cue overload and encoding-retrieval match orthogonally and found that higher encoding-retrieval match conditions could result in less efficient retrieval due to interference from overloaded (shared) cues. Their design is replicated in Experiment 1 and is described in more detail later.

Prior research has shown age differences in encoding specificity, cue reinstatement, and cue overload effects, and these differences may reflect different discriminative memory processes in older adults (OA). Compared with young adults (YA), OA have a tendency to rely on gist-based processing (Craik & Simon, 1980; Reder, Wible, & Martin, 1986). They often respond to a memory test stimulus on the basis of its general meaning as opposed to its precise surface form (e.g., Koutstaal & Schacter, 1997). This has been used as an explanation for data showing smaller encoding specificity effects in OA compared with YA (Puglisi, Park, Smith, & Dudley, 1988; Rabinowitz, Craik, & Ackerman, 1982), because OA may have deficits in forming specific memory traces. On the other hand, there are studies that show similar encoding specificity effects in young and older adults (Craik & Schloerscheidt, 2011; Park, Puglisi, Smith, & Dudley, 1987; Park, Puglisi, & Sovacool, 1984). Craik and Schloerscheidt (2011), for instance, showed that under intentional learning instructions, OA could benefit as much if not more than YA from the reinstatement of the study context at the point of retrieval (albeit in a recognition memory paradigm). The mixed literature in this area highlights the need for further research with the aim of developing a better understanding of how age interacts with the principles that govern retrieval.

Cue-overload effects have been linked to age deficits in inhibitory processing (cf. Hasher & Zacks, 1988), which may hinder the ability of OA to suppress interference from the competing activity evoked by overloaded cues. OA generally show an increase in the fan effect (e.g., Cohen, 1990; Gerard, Zacks, Hasher, & Radvansky, 1991; Radvansky, Zacks, & Hasher, 1996), where the number of concepts subsumed by a cue reduces the efficacy of that cue. Thus, responses to cues containing nondiagnostic information may be more problematic for OA as they are usually less able to direct their cognitive resources away from irrelevant information (cf. Zacks, Radvansky, & Hasher, 1996).

One of the issues complicating the interpretation of cue manipulations is the well-documented associative deficit observed in OA. In a nutshell, OA are disproportionately disadvantaged when memory performance depends on developing new associations between elements of information—such as between cues and TBR information (Chalfonte & Johnson, 1996; Naveh-Benjamin, 2000; Old & Naveh-Benjamin, 2008). It follows that in many cases, disproportionate effects of cuing manipulations in OA can be attributed to deficits in associating cue and target (Smyth & Naveh-Benjamin, 2016). Also, many studies have shown that prior knowledge can significantly modulate the interaction of age effects and cuing manipulations (see Umanath & Marsh, 2014, for a review). Information consistent with existing schemas (information aligned with knowledge and experience) is generally more memorable than information inconsistent with existing schemas (Alba & Hasher, 1983) and schematic memory effects are often more extreme in OA (e.g., Badham, Estes, & Maylor, 2012; Badham, Hay, Foxon, Kaur, & Maylor, 2016; Naveh-Benjamin, Hussain, Guez, & Bar-On, 2003; Smith, Park, Earles, Shaw, & Whiting, 1998). This latter pattern prompted us to include prior knowledge as a factor in Experiment 1 as any relationship between aging and discrimination processes could be modulated by prior knowledge. Finally, research in the area typically investigates the impact of encoding specificity, context reinstatement and cue-overload (or fan) in isolation. This means it is usually not possible to (a) examine any interaction between these factors, (b) assess their relative impact, and (c) examine any age-related effects on the discrimination process overall.

Our general aim was to explore how age might interact with the cue-based discrimination process. To do so, Experiment 1 calls upon Poirier et al.’s (2012) novel paradigm while also including a manipulation of prior knowledge. The paradigm involves training participants with respect to the relationship between cues and targets, with the aim of bringing accuracy of both YA and OA to ceiling, hence minimizing differences in encoding and associative learning between conditions and groups (something that can also be supported by prior knowledge); RT was used as the measure of cuing effects, as in Poirier et al. The training aspect of the task called upon in Experiment 1 had another advantage. It allowed us to ask whether most OA could be trained to perform as well as YA when it comes to associating multiple cues to memory targets. To anticipate, the answer is a qualified ‘yes’ as this was possible for more than 70% of the OA sample in the conditions studied here. Moreover, the extent of the training necessary to equate the YA and OA in terms of retrieving a target given a set of cues could be assessed. To our knowledge, there have been few attempts to determine how many study episodes are necessary to equate performances (but see Radvansky et al., 1996), a procedure that can be seen as an alternative measure of the associative deficit in OA. Experiment 2 examined the same memory-as-discrimination process but in a paradigm where accuracy was the main dependent variable (i.e., discrimination was assessed in the context of age-related associative deficits).

## Experiment 1

In Experiment 1, we manipulated the number of cues that were reinstated at test—essentially a manipulation of encoding-retrieval match—while simultaneously manipulating cue overload. Simply put, after learning cue–cue–target ensembles, participants could be presented with either one or two cues. The nature of the second cue was manipulated so that it would support discrimination in one case but not in the other, because in the latter it was associated with more than one target. What were the expected results? The fan effect studies mentioned above as well as the proposed age deficit in inhibition suggest that OA should be more affected by the introduction of overloaded cues (Radvansky et al., 1996). In the context of Experiment 1, this would predict that going from two discriminative cues to a condition where one of two cues is overloaded could be more detrimental to OA than to YA. The empirical findings on context reinstatement (i.e., Craik & Schloerscheidt, 2011; Park et al., 1987; Park et al., 1984) suggested no age-related effect—that is, a comparable benefit across age groups when the number of discriminative cues is increased—or perhaps a small age-related benefit. Thus, reinstating two discriminative cues compared with only one should have a beneficial effect—what is less clear is whether this effect will interact with age. If there is an age-dependent decrement when overloaded cues are introduced and no age-related effect when discriminative information is added, the overall pattern would then support the hypothesis that the discrimination process in OA is less efficient than in YA.

However, as mentioned above, associative deficits could also explain many of the prior cue-related findings; as we attempted to minimize their impact, precise predictions based on prior results are more uncertain. It was also more difficult to make precise predictions with respect to how schematic knowledge would interact with these other processes; prior research clearly suggests that schematic knowledge should help both learning and retrieval. What is less clear is whether this support could alleviate any deficits in the discrimination process as has been seen in other paradigms (e.g., Naveh-Benjamin et al., 2003).

### Method

#### Design

Participants studied triplets of pictures (two cues and a target); two of the pictures could be used in a later cued-recognition task where the third picture (the target) had to be identified. Targets had either two unique cues that were not shown with other targets, or one unique cue and one shared cue where the shared cue was shown with two targets (see Table 1 for example stimulus sets). This produced four possible cuing conditions at test: one unique cue, one shared cue, one unique plus one shared cue, and two unique cues. The overall design was Age (two levels: YA, OA) × Schematic knowledge (two levels: schema-consistent stimuli, schema-inconsistent stimuli) × Cuing condition (four levels: one unique cue, one shared cue, one unique plus one shared cue, and two unique cues).[Table-anchor tbl1]

#### Participants

Thirty-six YA (21 female) aged 18–27 years (*M* = 21.7, *SD* = 2.0) and 36 healthy OA (22 female) aged 64–91 years (*M* = 72.8, *SD* = 6.1) took part in the experiment. This excludes one of the YA and three OA who failed to follow instructions and five OA who were unable to proceed beyond the training phase of the experiment. YA were recruited from the University of Warwick and received either £6 or course credit. OA were all living independently and were recruited from an Age Study Volunteer Panel populated by local advertisements; they each received £10 toward their travel expenses.

YA and OA did not differ significantly in their years of education, *t* < 1 (*M*_YA_ = 15.25, *SD*_YA_ = 1.83; *M*_OA_ = 15.21, *SD*_OA_ = 3.36). To assess cognitive functioning, participants completed the Digit Symbol Substitution test from the Wechsler Adult Intelligence Scale—Revised (Wechsler, 1981) as a measure of processing speed, and the multiple choice part of the Mill Hill vocabulary test (Raven, Raven, & Court, 1988) as a measure of crystallized intelligence. The results were consistent with the literature (e.g., Salthouse, 2010): YA performed better than OA at the speed task, *t*(70) = 9.17, *p* < .001 (*M*_YA_ = 74.53, *SD*_YA_ = 10.93; *M*_OA_ = 51.61, *SD*_OA_ = 10.26), and OA performed better than YA at the vocabulary task, *t*(70) = 10.75, *p* < .001 (*M*_YA_ = 15.36, *SD*_YA_ = 3.77; *M*_OA_ = 24.47, *SD*_OA_ = 3.41).

#### Materials

Sets of related pictures (e.g., transport: boat, car, train and airplane) were used to create cue–cue–target triplets. These pictures could be integrated together with the help of existing schematic knowledge. The stimulus sets were drawn from six categories of items (clothing, furniture, nautical, stationery, tools, and transport), with four exemplars in each category. The stimuli were chosen so that certain exemplars could be applicable to two categories (e.g., *boat* might appear in a set based on transport or in a set based on nautical objects). Exemplars from these categories were also recombined to form memory sets for the condition that was inconsistent with participants’ schematic knowledge.

For the schema-consistent condition, the cue–cue–target triplet pictures were all related. For the schema-inconsistent condition, each cue–cue–target triplet was created from an exemplar from each of three different categories (excluding exemplars applicable to more than one category). For each condition, participants were shown three cue–cue–target triplets, with two of the triplets sharing a cue picture (see Table 1 for examples). Importantly, for any given participant, no pictures or categories were repeated between schema-consistent and schema-inconsistent conditions.

During encoding and retrieval, the pictures were shown in monochrome, using E-Prime 2 presentation software (Psychology Software Tools, Sharpsburg, PA) with a width and height of 300 pixels which corresponded to a width/height viewing angle of approximately 6.5°. Six different combinations of stimuli were generated, following the constraints outlined above. These were presented in two test orders (schema-consistent then schema-inconsistent or vice versa), resulting in 12 different versions of the experiment which were each used with three YA and three OA.

#### Procedure

The procedure replicated Poirier et al.’s (2012) Experiment 4 but with an additional learning phase and a modification to the one shared cue condition. The entire procedure was completed once with schema-consistent stimuli and once with schema-inconsistent stimuli by each participant.

Participants studied the cue–cue–target picture triplets during an encoding phase. The two cue pictures were shown at the top of the screen and the target picture was presented below them. Participants were informed that they should study the pictures and that they would later be shown one or two of the top pictures and would be asked to indicate the target picture with which they were originally shown. Each triplet was shown twice, producing six encoding trials, with the order of the image triplets randomized. Each trial randomly displayed a given cue on the left or right of the other cue. The triplets were displayed for 3.5 s on a plain white background with a 0.5-s plain white blank screen between trials.

#### Learning Phase I

Participants initially completed a separate learning-orientated memory task with feedback to encourage learning of all associations. Prior research with this paradigm showed that some participants associate the shared cue more strongly to one of its targets than to the other (Koutmeridou, Fowler, & Poirier, 2011). This could potentially confound the data as responses based on the shared cue could be faster for the dominant target and slower for the nondominant target, influencing the overall measure of RT. This initial memory task, which required knowledge of all the associations, was therefore conducted before moving on to the next phase of the experiment. Participants were presented with all five cues along the top of the screen (with numbers 1–5 below them) and one of the targets at the bottom of the screen. The task was to choose both cues that went with each target by pressing the number keys on the keyboard corresponding to the matching cues. If a correct response was made, the word ‘Correct!’ appeared for 1.5 s. If an incorrect response was made, the phrase ‘Incorrect! Answer should be:’ was displayed above the correct answer showing the study trial triplet again for 4 s, followed by a repeat of the test trial. This task continued until the participant had responded correctly to all triplets twice (a minimum of six trials). Following the successful completion of this initial task (see Learning Data I in the Results), participants moved on to the second learning phase.

#### Learning Phase II

The second memory task consisted of re-presentation of the encoding phase followed by a retrieval phase where participants chose a single target corresponding to one or two cues provided on each test trial. Participants were shown four trials from each of the four cuing conditions (one unique cue, one shared cue, one unique plus one shared cue, and two unique cues) producing a test phase of 16 trials. Cue positions were again randomized on each trial. For the one unique condition, two of the four trials used the two unique cues from the study triplet with two unique cues. For the other two trials, the unique cues were one from each of the study triplets consisting of one unique plus one shared cue. When only one cue was shown, a small black square occupied the space of the missing cue. For each test trial, all three targets were shown along the bottom of the screen and below each target from left to right were displayed the digits 1, 2, and 3. Participants were instructed to place the first three fingers of their right hand on the keys 1 through 3 and to press the number corresponding to the target that was originally displayed with the cue(s). Participants were instructed to respond as accurately as possible. The order of the three targets was random but they remained in the same position on every trial for a given participant. Across the counterbalancing conditions, the ‘two-unique’ target was placed equally often in the 1st, 2nd, or 3rd position. Additionally, for a given participant, the ‘two-unique’ target occupied the same position in the schema-consistent and schema-inconsistent conditions. For the one shared cue condition, two answers would have been possible, but one of the answers was removed from the screen. This was arranged such that the shared cue was used equally often for the two targets to which it was associated. This was done to further discourage participants from choosing a dominant target for the shared cue and was another manipulation not applied in Poirier et al. (2012). The encoding and retrieval phases were repeated until a participant had scored 75% correct (for all four cue conditions considered separately) on two consecutive attempts or until six encoding-retrieval blocks had been completed (see Learning Data II in the Results).

#### Memory-as-discrimination test

Following the second learning phase, participants carried out a longer, critical version of the test section from Learning Phase II consisting of 48 trials (12 for each cue condition). In this critical version, participants were additionally instructed to respond as quickly as possible. Test trial order was randomized for each participant.

Once the whole procedure was completed with one level of schema consistency, it was then repeated with the other. Participants were allowed to rest for as long as they wanted between these two conditions.

### Results

#### Learning Data I

**Figure 1 fig1:**
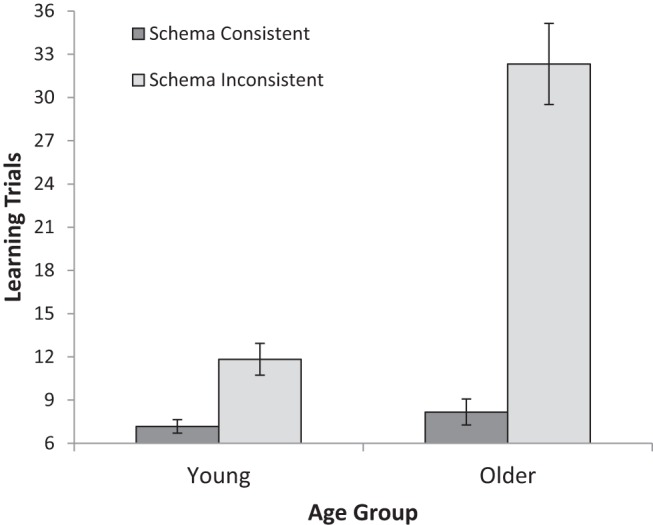
Mean number of learning trials required to reach 100% accuracy for six trials in a row (minimum = 6) for young and older adults and for schema consistent and schema inconsistent conditions in Experiment 1. Error bars are ± 1*SE*.

The number of learning trials required to achieve correct responses to all three picture combinations twice in a row was calculated separately for schema-consistent and schema-inconsistent picture conditions (see Figure 1 for means). A 2 (Age: YA, OA) × 2 (Schema consistency: consistent, inconsistent) mixed ANOVA showed superior learning in YA compared with OA, *F*(1, 70) = 38.54, *MSE* = 107.94, *p* < .001, η_*p*_^2^ = .36. Schema consistent groupings were easier to learn than schema inconsistent groupings, *F*(1, 70) = 99.93, *MSE* = 74.88, *p* < .001, η_*p*_^2^ = .59. These two factors interacted, with age deficits much larger for schema inconsistent groupings than for schema consistent groupings, *F*(1, 70) = 45.70, *MSE* = 74.88, *p* < .001, η_*p*_^2^ = .40.[Fig-anchor fig1]

#### Learning Data II

Table 2 shows the number of participants reaching the criterion of scoring 75% correct for two consecutive learning blocks for the second learning task (note that some participants failed to reach criterion even after six blocks). Again, OA required more training than did YA, particularly in the schema inconsistent condition. Significantly greater numbers of YA than OA needed only the minimum number of blocks in both the schema consistent condition, χ^2^(1) = 4.13, *p* = .042, and schema inconsistent condition, χ^2^(1) = 5.57, *p* = .018.[Table-anchor tbl2]

#### Memory-as-discrimination test

As in Poirier et al. (2012), participants who did not achieve at least 60% correct in all four cuing conditions for both schema consistent and schema inconsistent conditions were excluded. Of the initial 36 YA and 36 OA, 33 YA and 26 OA met these criteria.^1^ By design, the primary measure of performance for this experiment was RT. To simplify the reporting of results, the accuracy data are summarized in Appendix A and the main analyses are reported on the RT data. In brief, the accuracy data were qualitatively similar to the RT data in that higher accuracy corresponded to faster responses (i.e., there were no speed–accuracy trade-offs).

**Figure 2 fig2:**
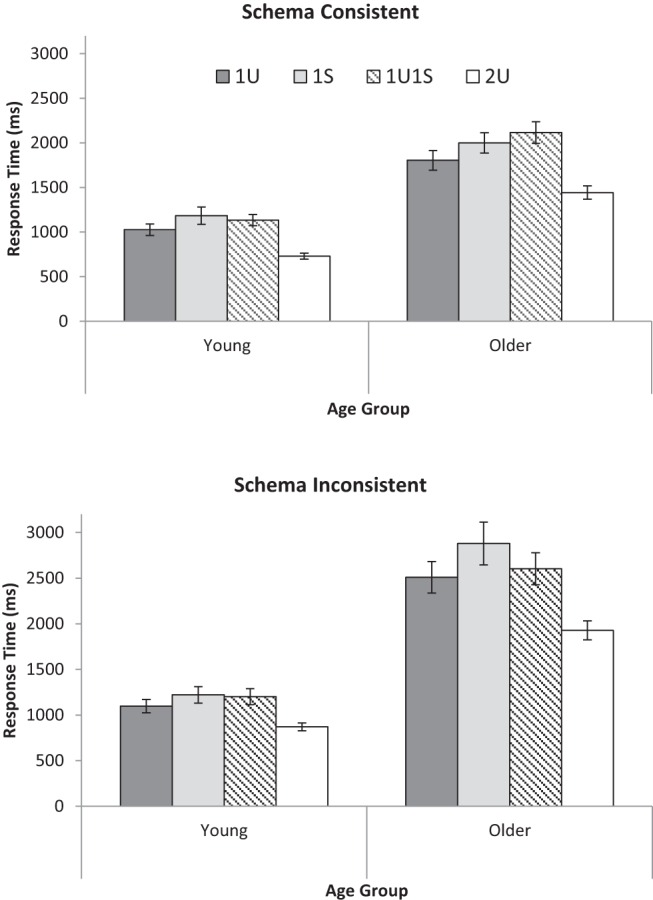
Mean correct response times in milliseconds (ms) for young and older adults for schema consistent (top) and schema inconsistent (bottom) stimuli and for four cuing conditions: one unique cue (1U), one shared cue (1S), one unique plus one shared cue (1U1S), and two unique cues (2U) in Experiment 1. Error bars are ± 1 *SE*.

A 2 (Age: YA, OA) × 2 (Schema consistency: consistent, inconsistent) × 3 (Cuing condition: one unique, one unique plus one shared, two unique) mixed ANOVA was conducted on the RT data for correct responses for each participant and each condition (see Figure 2 for means). Median RTs were used to reduce the influence of any extreme RT (following Poirier et al., 2012) because of the relatively small number of trials available to estimate RT in each condition (see Radvansky et al., 1996, for a similar strategy in studying the fan effect). Note that the one shared cue condition was excluded because there were fewer responses available on screen which would artificially influence RTs. YA responded faster than OA, *F*(1, 57) = 112.13, *MSE* = 8.7 × 10^5^, *p* < .001, η_*p*_^2^ = .66. Responses to schema consistent groupings were faster than responses to schema inconsistent groupings, *F*(1, 57) = 35.79, *MSE* = 2.58 × 10^5^, *p* < .001, η_*p*_^2^ = .39. Age and schema consistency interacted, *F*(1, 57) = 18.46, *MSE* = 2.58 × 10^5^, *p* < .001, η_*p*_^2^ = .25, with OA showing greater slowing for schema inconsistent groupings than for schema consistent groupings. There was a main effect of cuing condition, *F*(2, 114) = 55.94, *MSE* = 2.98 × 10^5^, *p* < .001, η_*p*_^2^ = .50, and an interaction between age and cuing condition, *F*(2, 114) = 4.83, *MSE* = 2.98 × 10^5^, *p* = .010, η_*p*_^2^ = .08, which is explored below. There were no other effects (remaining *F*s < 1.67, *p*s > .19).[Fig-anchor fig2]

The different cuing conditions were further investigated; for simplicity, this paragraph only reports effects involving cuing condition. To assess memory as discrimination, a 2 (Age: YA, OA) × 2 (Schema consistency: consistent, inconsistent) × 2 (Cuing condition: one unique, one unique plus one shared) mixed ANOVA was conducted. Responses to one unique cue were faster than responses to one unique plus one shared cue, *F*(1, 57) = 10.30, *MSE* = 1.33 × 10^5^, *p* = .002, η_*p*_^2^ = .15, supporting the memory-as-discrimination view. There were no interactions involving cuing condition (*F*s < 1.24, *p*s > .27). A similar ANOVA was conducted with cuing conditions of one unique cue and two unique cues to assess effects of encoding-retrieval match. Responses to two unique cues were faster than responses to one unique cue, *F*(1, 57) = 61.48, *MSE* = 1.28 × 10^5^, *p* < .001, η_*p*_^2^ = .52, demonstrating an advantage for greater encoding-retrieval match. Note that this was despite the requirement to process two cues rather than only one. There was an interaction between age and cuing condition, *F*(1, 57) = 5.05, *MSE* = 1.28 × 10^5^, *p* = .029, η_*p*_^2^ = .08, and also a triple interaction between age, schema consistency, and cuing condition, *F*(1, 57) = 6.40, *MSE* = 4.85 × 10^4^, *p* = .014, η_*p*_^2^ = .10, attributable to the speedup between one and two cues being greatest for OA with schema-inconsistent stimuli. Finally, a similar ANOVA was conducted with the cuing conditions of one unique plus one shared cue and two unique cues to assess the effects of cue overload. Responses to two unique cues were faster than responses to one unique plus one shared cue, *F*(1, 57) = 84.86, *MSE* = 1.86 × 10^5^, *p* < .001, η_*p*_^2^ = .60. This interacted with age, *F*(1, 57) = 7.40, *MSE* = 1.86 × 10^5^, *p* = .009, η_*p*_^2^ = .12, with OA showing greater effects of cue overload than YA.

#### Proportional slowing?

In considering these RT effects, it is important to take into account generalized slowing in old age (Cerella, 1990; Myerson, Hale, Wagstaff, Poon, & Smith, 1990; Salthouse, 1996). Aging generally leads to multiplicative rather than additive effects on RTs, which complicates the interpretation of absolute age differences between conditions (see Maylor, Schlaghecken, & Watson, 2005; Verhaeghen, 2011). Thus, to assess whether any age-related differences in RT effects were larger than expected purely on the basis of proportional slowing, a logarithmic transformation was applied to the present data before repeating the above RT analyses. For the initial 2 × 2 × 3 ANOVA (see Appendix B for details), the interaction between age and schema consistency remained significant, *p* < .01, and was also significant in all the follow-up ANOVAs. In contrast, all interactions between age and cuing conditions were no longer significant (all *F*s < 1), indicating comparable (proportionate) effects of cuing in YA and OA.

### Discussion

Experiment 1 successfully replicated prior research showing faster responses to a single unique cue compared with responses to a unique cue plus a shared cue in a cued-recognition memory task (Poirier et al., 2012); this applied equally to YA and OA. An additional cue increased the degree of encoding-retrieval match but when it had little diagnostic value (i.e., it was associated to multiple targets) performance was hindered. Therefore, the notion that higher encoding-retrieval match always results in better memory is not supported by the current data, which are better viewed under a memory-as-discrimination hypothesis (Capaldi & Neath, 1995; Craik & Jacoby, 1979; Eysenck, 1979; Goh & Lu, 2012; Hunt, 2003; Nairne, 2002; Poirier et al., 2012; Surprenant & Neath, 2009). Importantly, the slowing induced in the one unique plus one shared cue condition cannot be attributed to having two cues to process as, relative to the single unique cue condition, processing two unique cues reduced RT.

In absolute terms, OA benefitted more from greater encoding-retrieval match than did YA and showed greater levels of cue overload; however, these effects appeared to be due to general age-related slowing (i.e., generally slower responses led to larger differences between cuing conditions). In contrast, schema consistent stimuli successfully alleviated the age deficit in associative memory compared with schema inconsistent stimuli, even after controlling for general slowing in old age (cf. Badham et al., 2012; Naveh-Benjamin et al., 2003).

An additional noteworthy aspect of the current data is the age difference in learning schema consistent and schema inconsistent groups of stimuli. OA were particularly poor at learning schema inconsistent stimuli yet almost as good as YA at learning schema consistent stimuli, resulting in a remarkably large age by schema consistency interaction (Figure 1; see also Table 2). It seems likely that the schema-inconsistent groupings were particularly difficult for OA to learn because the cues often were categorically related to targets with which they were not presented (e.g., in Table 1, *chair* was a cue for the target *lifebuoy* but *bed* was also a target). Age deficits in memory can be reduced by schema consistent stimuli but also exacerbated by schema inconsistent stimuli (Umanath & Marsh, 2014) so the current stimuli generated a striking contrast in age differences between conditions. As with the memory-as-discrimination test data, the learning data are aligned with studies showing alleviation of age-related associative deficits under conditions of higher schematic support (e.g., Naveh-Benjamin et al., 2003). They also extend these findings by showing that difficulties with learning new associations are clearly also found within situations offering multiple learning trials and calling upon more than one learning task. Such findings are relevant with respect to learning and memory in typical environments as they suggest that a large number of learning trials are probably necessary to eliminate age-related deficits in learning new materials. The suggestion is that there are numerous situations—for example, new phones, new phone numbers, new PINs, new postcodes, new appliances with different controls—where multiple learning attempts will be necessary before performance asymptotes. Moreover, the results are very clear in showing that if learning can rely on prior schematic knowledge, age-related deficits in memory performance are very significantly reduced.

Overall, Experiment 1 supports a memory-as-discrimination hypothesis, which has not been previously assessed with data from OA. Our findings suggest that the negative effects of cue overload can outweigh benefits of increased encoding-retrieval match in both age groups; the results of Experiment 1 hence suggest age-invariance with respect to manipulations of cue–target distinctiveness.

Importantly, however, in many circumstances episodic retrieval cannot benefit from a large number of retrieval attempts. In these situations, OA are at disadvantage relative to YA at least in part because of the age-related difficulty in calling upon newly formed associations. In Experiment 1, discrimination was found to be age-invariant but this was in the context of a task where training minimized any group differences in learning the cue–target relationships. In Experiment 2, we examine the influence of age on the discrimination process without these equating procedures. Memory accuracy in a cued-recall task was used to test predictions derived by considering both the age-related associative deficit and the memory-as-discrimination view.

## Experiment 2

This experiment is conceptually similar to that of Goh and Lu (2012) where encoding-retrieval match was manipulated orthogonally to a manipulation of cue overload. As in Goh and Lu, the task was cued recall; however, here we added a context processing task at encoding which would be used to manipulate encoding-retrieval match and cue overload. In the Goh and Lu (2012) experiments, the overloaded cue was not included in the actual study phase but relied on previously established (but unstudied) cue–target links; these strategies may have contributed to reducing the impact of the manipulation.

Because this experiment relies on a cued-recall task involving a unique study trial for each pair of TBR items, it is important to consider the potential impact of the associative deficit in OA (Old & Naveh-Benjamin, 2008). A straightforward assumption is that an associative deficit will reduce—to paraphrase Tulving (1983)—the compatibility of the cue and the encoded information. This could have a number of effects, and to consider them adequately it is helpful to call upon a simple formalism used to summarize the ideas imbedded in the memory-as-discrimination view (Goh & Lu, 2012; Nairne, 2002; Poirier et al., 2012). In effect, this is a choice rule or sampling model often incorporated in memory and categorization models (Henson, 1999; Nairne, 2001, 2002; Nosofsky, 1986). This choice rule states that the probability that a particular event, *E*_1_, will be retrieved from memory depends on how well a cue, *C*_1_, matches (s for similarity) the target *E*_1_ to the exclusion of other retrieval candidates (*E*_2_, *E*_3_, . . . *E*_n_), as follows:

$$P_{r}\left(E_{1}|\;C_{1}\right)=\;\frac{s(E_{1},C_{1})}{\sum [s(E_{1},C_{1}),\;. . .,s(E_{n},\;C_{1})]}$$ 1

As can be deduced from (1), any estimation of correct recall probability (*P_r_*) depends on the encoding-retrieval match, expressed in the numerator as the similarity (s) between the cue *C*_1_ and that target *E*_1_, and also on cue overload—here represented in the denominator by the summed similarity between the cue and all the items in the retrieval set (including the target).

Here we made the assumption that an associative deficit would impact the probability that the cue and target would be sufficiently compatible for this choice rule to operate—simply put, poor associative memory would lead to the target-cue relationship not being established. The result would be that the encoded information would not involve the necessary binding between cues and TBR information. As a consequence, *E*_1_ in equation (1) above would not involve the actual TBR target, leading to a zero probability of recall. The implication is that in the baseline one unique condition, there will be a larger number of trials for the OA where no response will be generated, leading to a drop in performance.

However, importantly, it is also reasonable to assume that poor associative memory would mean the links between cues and competing candidates would also be less potent. As a consequence, OA would often have a smaller denominator in the choice rule above as overloaded cues would not systematically elicit their associated targets. More generally, weak associative links could be assumed to reduce the denominator set size for all retrieval conditions. Importantly, this analysis predicts positive consequences; that is, when a cue does elicit a response there would be a higher probability of correct recall as the interfering information is less likely to be part of the retrieval set. When only one unique cue is provided, then the negative impact of the associative deficit is likely to mask any beneficial effect of a reduced set of competitors. However, for OA, going from one to two unique cues should have more impact than for YA; on the one hand, there should be an increased probability that one of the two unique cues will elicit the target, whereas on the other, it is likely that the number of competitors included in the search set will be smaller for OA than for YA; the net result would be more change from one unique to two unique cues for OA.

Also, going from a unique cue to a unique plus a shared cue should be less disruptive for OA, as the associations to the interfering items are not as potent. These two examples lead to predicting interactions between age and the specific comparisons mentioned. Moreover, the analysis leads to the prediction that going from one to two cues in the OA, overall, should prove more positive than it is for the YA (i.e., an interaction between age and encoding-retrieval match is predicted).^2^

### Method

#### Design

Participants studied pairs of words and were later required to recall the right word of each pair when presented with the left word of each pair. Cue overload and encoding retrieval match were manipulated. The word-cue was always uniquely associated to the TBR target. During study, before the presentation of each word pair, participants completed a ‘spot the difference’ task where they had to identify a small difference between two otherwise identical images. Word pairs were then presented on top of the images; this made it possible to use the images as contextual cues for the target words. Some images were used as backgrounds for four word pairs and served as shared (overloaded) cues. Some images were unique to a given word pair and served as unique cues. During retrieval, cue words were presented alone (low encoding-retrieval match) or on top of the image with which they were encoded (high encoding-retrieval match). The overall 2 × 2 × 2 design was: Age (YA, OA) × Encoding condition (unique/shared images) × Retrieval condition (low/high encoding-retrieval match).

#### Participants

Thirty YA (16 female) aged 20–27 years (*M* = 20.9, *SD* = 1.4) and 31 healthy OA (16 female) aged 65–83 years (*M* = 71.8, *SD* = 5.1) took part in the experiment. OA were all living independently and were recruited through the University of the Third Age, the Warwick Arts Centre and personal contact. No compensation was offered for participation. YA and OA did not differ significantly in their years of education, *t* < 1 (*M*_YA_ = 16.22, *SD*_YA_ = 0.74; *M*_OA_ = 16.24, *SD*_OA_ = 3.70). The same speed and vocabulary tasks were completed as reported in Experiment 1: YA performed better than OA at the speed task, *t*(59) = 6.67, *p* < .001 (*M*_YA_ = 69.97, *SD*_YA_ = 14.99; *M*_OA_ = 47.55, *SD*_OA_ = 11.02), and OA performed better than YA at the vocabulary task, *t*(44.4) = 4.44, *p* < .001 (*M*_YA_ = 19.43, *SD*_YA_ = 2.94; *M*_OA_ = 24.71, *SD*_OA_ = 5.89).

#### Materials

**Figure 3 fig3:**
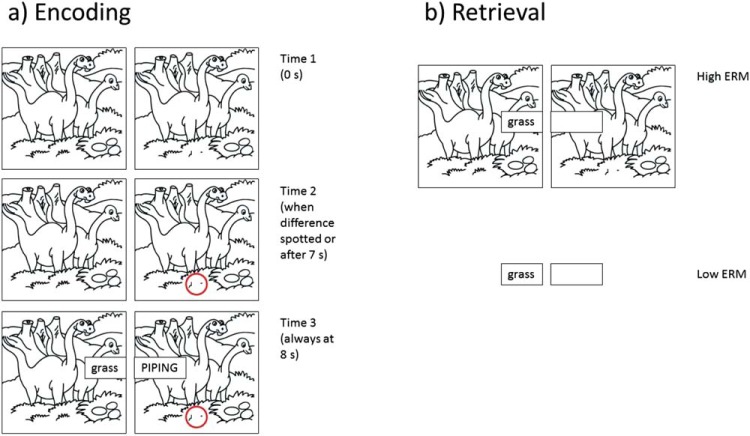
Example of encoding and retrieval items from Experiment 2. (a) Participants studied a spot-the-difference image set; when they spotted the difference (or after 7 s, whichever was the shorter) the difference was highlighted with a red circle. At 8 s, the to-be-remembered word pair appeared for a duration of 6 s. (b) In the high encoding-retrieval match (ERM) condition (top), the cue word was presented with its corresponding background. In the low ERM condition (bottom), the cue word was presented alone (a participant would only see one of these retrieval conditions for a given word pair). See the online article for the color version of this figure.

The experiment was programmed with a combination of Html5, JavaScript, and Perl and was displayed in a web browser on a computer. Participants completed a practice trial and nine experimental trials. Each trial involved the presentation of eight image sets, with participants attempting to spot a difference between the images before studying a corresponding pair of words (an example is depicted in Figure 3a). Black and white hand-drawn images depicting simple scenes were taken from a puzzle book (Benders, 2007); permission for their use was obtained from the publisher. To create a spot-the-difference task, two images were presented side by side which were identical apart from a small area hidden by a white box in the right image. The TBR word pairs were shown on top of the images after a delay (see below and Figure 3a), the left word being a noun that was related to the difference between the two spot-the-difference images. The left word was presented in lower case and would later be the cue word in the cued-recall test. The right word of each pair was presented in upper case and would later be the target word in the cued-recall test. The right target words were randomly selected from a total of 96 two-syllable nouns from Friendly, Franklin, Hoffman, and Rubin (1982), which had a mean concreteness rating of 5.32 (on a 7-point scale). No words or images were repeated across trials.[Fig-anchor fig3]

To generate shared/overloaded cues, across the eight pairs in a trial, one of the spot-the-difference image sets was used four times but with a separate difference in each case. For the remaining four pairs, four different spot-the-difference image sets were used. Therefore, one image set was associated to four separate word pairs while the remaining four image sets were each associated to just a single word pair.

At retrieval, participants were shown the left word of each word pair and were asked to recall the right word (see Figure 3b for an example). Half of the retrieval items had high encoding-retrieval match where the original spot-the-difference image sets were presented with the cues in the same format as at encoding. The remaining half of the retrieval items presented the cue words in isolation. Retrieval cues were presented in a random order.

#### Procedure

Participants initially completed a practice trial. Following this, they completed nine trials with a rest break of at least two minutes after the first four. Finally they completed the speed and vocabulary tests described previously.

For the spot-the-difference task, participants were required to point at the difference between the displayed images and if successful, the experimenter then pressed a button which made an empty red circle appear around the difference on the right image. The display then remained until the TBR word pair was presented, 8 s after the images first appeared. If no difference was spotted, the red circle would appear automatically after 7 s and one second later the TBR word pair appeared with the left word over the left image and the right word over the right image for a total duration of 6 s. All images and word pairs were presented in a random order and there was a 0.5-s blank screen after the presentation of each pair. Immediately after eight image sets and eight word pairs were studied, participants completed the retrieval phase for that trial. At retrieval, the left word of each word pair was displayed either with or without the original background spot-the-difference image set (depending on the encoding-retrieval match condition). Participants were instructed to say out loud the target word that was originally presented to the right of that cue. The experimenter then typed their response into the computer. Participants were allowed to move on to the next cue if they could not remember the target word.

### Results

#### Spot-the-difference performance

The proportion of differences that were spotted was calculated for each participant and background type (see Table 3 for means). A 2 (Age: YA, OA) × 2 (Image type: unique, shared) mixed ANOVA showed better performance in YA compared with OA, *F*(1, 59) = 93.43, *MSE* = 0.03, *p* < .001, η_*p*_^2^ = .61, similar performance for unique image sets compared with shared image sets, *F* < 1, and a marginal interaction, *F*(1, 59) = 3.27, *MSE* = 0.01, *p* = .076, η_*p*_^2^ = .05, with age deficits numerically larger for shared image sets than for unique image sets.[Table-anchor tbl3]

#### Memory performance

**Figure 4 fig4:**
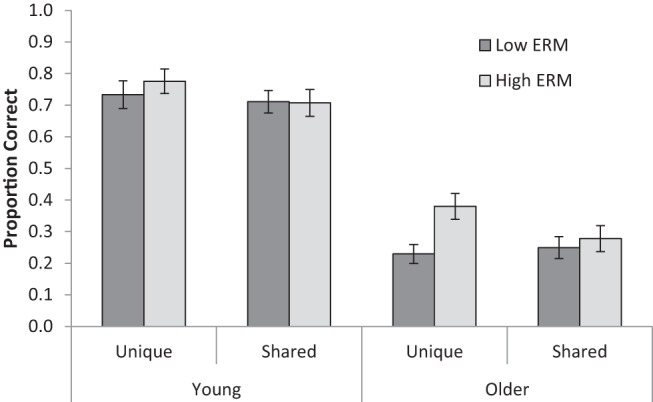
Accuracy for young and older adults’ cued recall performance for word pairs encoded with unique or shared spot-the-difference images (low or high cue overload, respectively) and for low and high encoding-retrieval match (ERM) retrieval conditions in Experiment 2. Error bars are ±1*SE*.

A 2 (Age: YA, OA) × 2 (Encoding condition: unique/shared images) × 2 (Retrieval condition: low/high encoding-retrieval match) mixed ANOVA was conducted on the proportion of words successfully recalled (see Figure 4 for means). YA performed better than OA, *F*(1, 59) = 80.99, *MSE* = 0.15, *p* < .001, η_*p*_^2^ = .58. Memory was superior when word pairs were encoded with unique compared with shared background images, *F*(1, 59) = 10.68, *MSE* = 0.01, *p* = .002, η_*p*_^2^ = .15, suggesting an overall cue-overload effect. Memory was superior when images were present at retrieval, *F*(1, 59) = 16.09, *MSE* = 0.01, *p* < .001, η_*p*_^2^ = .21, indicating better memory for the high compared with the low encoding-retrieval match conditions.[Fig-anchor fig4]

These main effects were qualified by two significant interactions. The mnemonic benefit of higher encoding-retrieval match interacted with age, *F*(1, 59) = 6.66, *MSE* = 0.01, *p* = .012, η_*p*_^2^ = .10. Follow-up tests showed no significant effect of encoding-retrieval match in YA, *t*(29) = 1.60, *p* = .120, but a significant effect of encoding-retrieval match in OA, *t*(30) = 3.73, *p* < .001.

There was also an interaction between encoding and retrieval conditions, *F*(1, 59) = 10.95, *MSE* = 0.01, *p* = .002, η_*p*_^2^ = .16. Follow-up tests showed that the effect of higher encoding-retrieval match was only present when the reinstated background was unique, *t*(60) = 4.35, *p* < .001, and not when the reinstated background was shared, *t* < 1. Therefore, in line with the memory-as-discrimination view, increased encoding-retrieval match was only beneficial when the additional cues afforded more discriminability. Finally, there was no triple interaction between age, encoding condition and retrieval condition, *F*(1, 59) = 2.21, *MSE* = 0.01, *p* = .142, η_*p*_^2^ = .04.

### Discussion

Experiment 2 again provides support for the memory-as-discrimination view (Goh & Lu, 2012; Nairne, 2002; Poirier et al., 2012) and shows that its predictions are supported with a new paradigm and with OA. YA showed better memory performance than OA, shared/overloaded cues were less helpful than unique cues, and high encoding-retrieval match was beneficial to memory relative to low encoding-retrieval match. Importantly, the data showed that increasing encoding-retrieval match can lead to no change in performance when the said increase does not improve the capacity of the cue constellation to specifically identify a target. These results are aligned with those of Goh and Lu (2012), who similarly found that nondiscriminative cues did not aid memory accuracy.

The current data also showed a dramatic overall age-related deficit in performance as well as a larger effect of encoding-retrieval match in OA compared with YA. The decrement in the performance of OA certainly seems to suggest that providing a unique cue did not frequently lead to a successful retrieval attempt. However, as expected, increasing the number of cues had a more favorable effect for OA than for YA. These findings are in line with what was expected assuming the associative deficit in OA would (a) lead to fewer retrievals in OA, and (b) would reduce the impact/size of the search set included in the denominator of Equation (1).

## General Discussion

We conducted two experiments that provided evidence in support of the memory-as-discrimination view with both YA and OA. In Experiment 1, a training procedure equated memory accuracy across YA and OA and examined how changes in cue discrimination power affected RT. In Experiment 2, there was no training and a cued recall task was called upon to examine the predictions derived from combining a memory-as-discrimination analysis with a consideration of the age-related deficit in associative memory (Old & Naveh-Benjamin, 2008).

Experiment 1 showed overall age deficits in the speed of retrieval processes, even when the associations between cues and targets were well enough established to support equivalent retrieval accuracy across age. Importantly, the balance of discriminative processing was qualitatively similar in the two age groups, which both showed effects of memory-as-discrimination, encoding-retrieval match, and cue overload. This demonstrated that the relative influence of cue–target compatibility and competing retrieval candidates was not influenced by the aging process after controlling for accuracy. This was not what was expected based on prior fan-effect studies, which showed greater cue overload in older adults (e.g., Radvansky et al., 1996). However, when these conclusions were based on RTs (Cohen, 1990; Gerard et al., 1991), there was no control for proportional slowing in older adults, in contrast to the current study.

Experiment 1 also showed considerable age differences in the amount of training necessary to equate retrieval accuracy, confirming the importance/impact of the age-related associative deficit (cf. Old & Naveh-Benjamin, 2008). Moreover, schematic support had a disproportionately beneficial effect on associative memory in OA relative to YA (Badham et al., 2012; Naveh-Benjamin et al., 2003).

In Experiment 2, there was no special procedure to equate associative memory in YA and OA and accuracy in a cued recall task was the dependent measure; the second experiment involved manipulations of discrimination power that were conceptually similar to the ones implemented in Experiment 1. We offered a number of predictions based on an analysis that integrated a simple model of the memory-as-discrimination view and some assumptions as to the impact of the age-related associative deficit. These predictions were supported: Performance for OA was very much lower than for YA and the expected interaction between encoding-retrieval match and age was obtained. Again, both age groups produced data in support of the memory-as-discrimination view.

Generally, this positive age-related impact of encoding-retrieval match is in line with prior reports suggesting that reinstatement benefits OA (e.g., Craik & Schloerscheidt, 2011). Importantly, however, the apparent age-related benefit from encoding-retrieval match is not viewed as an effect of match per se. The analysis that led to the predictions suggests that the benefit of a higher number of cues for OA is produced by the interplay of three factors: (a) presenting two unique cues leads to an increase in the probability of correct retrieval relative to a single unique cue, (b) presenting a unique plus a shared cue does not lead to an improvement in said probability, and (c) the associative deficit in OA will interact in predictable ways with (a) and (b). Specifically, the assumption was that the associative deficit would reduce the impact of competitors in the retrieval process; as a result, an increase in diagnostic information would have more impact for OA than for YA. Moreover, the same decrease in the retrieval competition would reduce the impact of the shared cue. The net result of these combined factors was that going from one to two cues should have more positive consequences for OA than for YA (or if the overall effect had been negative, then this negative impact would have been reduced in OA). This may seem to contradict the age-related inhibition deficit discussed previously (Hasher & Zacks, 1988) in that the overall prediction is that YA will show more impact of irrelevant information. However, our prediction relied on the assumption that the associations between cues and irrelevant information are not as well formed for OA. In Experiment 1, age differences in associative memory were minimized (as is typically the case in fan-effect studies) and the results did show an age-related decrement associated to overloaded cues. However, this age-dependent effect was not robust enough to remain when controls for proportional slowing were implemented.

Overall, the memory-as-discrimination analysis highlights that the observed impact of encoding-retrieval match completely depends on both the cue–target and the cue-competitor relationships. How these factors impact aging depends on how the associative deficit interacts with these all-important relationships between the cue constellation and the competing retrieval candidates.

## Figures and Tables

**Table 1 tbl1:** Example Categories and Pictures Used to Produce Cues and Targets in Schema Consistent and Schema Inconsistent Memory Sets in Experiment 1

Condition^a^	Category	Cue 1	Cue 2	Target
Schema consistent	Furniture (F)	bed	chair	wardrobe
	Transport (T)	**boat**	train	airplane
	Nautical (N)	**boat**	lighthouse	lifebuoy
Schema inconsistent	F + T + N	chair	car	lifebuoy
	F + T + N	**airplane**	table	lighthouse
	F + T + N	**airplane**	snorkel set	bed
*Note.* Bold cues are shared across two memory sets. The experiment used pictures but verbal labels are used here for clarity and copyright reasons.				
^a^ No participant saw the same stimuli in both conditions; the table shows an example of matched schema consistent and schema inconsistent stimuli shown to separate participants.				

**Table 2 tbl2:** Number of Participants Reaching Criterion After Completing 2 to 6 Learning Blocks (or Failing to Reach Criterion) in the Second Learning Task for Young and Older Adults and for Schema Consistent and Schema Inconsistent Conditions in Experiment 1

		Learning blocks					
Condition	Age	2	3	4	5	6	Failed
Schema consistent	Young	32	0	1	0	1	2
	Older	25	3	4	2	1	1
Schema inconsistent	Young	22	6	3	0	2	3
	Older	12	5	3	2	2	12

**Table 3 tbl3:** Mean (and Standard Deviation) Proportion of Differences Spotted for Young and Older Adults for Unique and Shared Image Sets in Experiment 2

Age	Unique image set	Shared image set
Young	.647 (.107)	.680 (.135)
Older	.368 (.132)	.355 (.150)

**Table A1 tbl4:** Mean (and Standard Deviation) Proportion Correct Accuracy for Young and Older Adults, for Schema Consistent and Schema Inconsistent Stimuli, and for All Four Cuing Conditions: One Unique Cue (1U), One Shared Cue (1S), One Unique Plus One Shared Cue (1U1S), and Two Unique Cues (2U) in Experiment 1

		Cuing condition			
Condition	Age	1U	1S	1U1S	2U
Schema consistent	Young	.987 (.030)	.960 (.081)	.972 (.045)	.985 (.033)
	Older	.990 (.027)	.993 (.023)	.968 (.048)	1.000 (.000)
Schema inconsistent	Young	.977 (.038)	.977 (.038)	.967 (.059)	.992 (.024)
	Older	.955 (.089)	.933 (.103)	.946 (.084)	.987 (.045)

## Footnotes

1 In both age groups, but particularly in the older group, more participants were lost who performed the tasks in the schema order inconsistent then consistent (2 YA; 8 OA) than consistent then inconsistent (1 of the YA; 2 OA). Including order as a factor in these ANOVAs produced interactions between schema consistency and order indicating practice effects such that participants’ responses were faster in the task they performed second. Note that the higher proportion of OA remaining in these analyses with the schema consistent then inconsistent order means that the observed age by schema consistency interactions are actually *underestimates* of the true effects. Importantly, the conclusions resulting from Experiment 1 remain unchanged when excluding the poorest 10 YA so that the same number of YA and OA were excluded.

2 Note, this is only true if the overall effect of encoding-retrieval match (going from one to two cues) is positive. If it is negative (i.e., a large negative contribution from one unique plus one shared cue) then the prediction would be that this negative effect should be reduced for OA.

## A Analysis of Accuracy in the Memory-as-Discrimination Test of Experiment 1

A 2 (Age: YA, OA) × 2 (Schema consistency: consistent, inconsistent) × 3 (Cuing condition: one unique, one unique plus one shared, two unique) mixed ANOVA was conducted on the accuracy data (see Table A1 for means; note that the one shared cue condition was not included in the ANOVA because it was not strictly comparable with the other cuing conditions, having fewer responses available on screen). There was no main effect of age, *F* < 1. Responses to schema consistent groupings were more accurate than to schema inconsistent groupings, *F*(1, 57) = 4.60, *MSE* = 0.003, *p* = .036, η_*p*_^2^ = .08. There was a marginal interaction between age and schema consistency, *F*(1, 57) = 2.99, *MSE* = 0.003, *p* = .089, η_*p*_^2^ = .05, with OA performing slightly better than YA with schema consistent stimuli, but worse than YA with schema inconsistent stimuli. There was a main effect of cuing condition, *F*(1.78, 101.42) = 11.46, *MSE* = 0.002, *p* < .001, η_*p*_^2^ = .17. Post hoc tests indicated that accuracy was significantly higher in the two unique condition relative to the one unique condition, *t*(58) = 2.68, *p* = .010, and to the one unique plus one shared condition, *t*(58) = 4.03, *p* < .001; responses were also more accurate in the one unique than in the one unique plus one shared condition, *t*(58) = 2.35, *p* = .022. Finally, there was also a marginal interaction between schema consistency and cuing condition, *F*(2, 114) = 2.52, *MSE* = 0.001, *p* = .085, η_*p*_^2^ = .04, suggesting somewhat larger cuing condition effects for schema-inconsistent stimuli. There were no other interactions (*F*s < 1.33, *p*s > .267).[Table-anchor tbl4]

## B Analysis of Natural-Logarithm Transformations of Response Times in the Memory-as-Discrimination Test of Experiment 1

A 2 (Age: YA, OA) × 2 (Schema consistency: consistent, inconsistent) × 3 (Cuing condition: one unique, one unique plus one shared, two unique) mixed ANOVA was conducted on the RT data after transformation using natural logarithms. YA responded faster than OA, *F*(1, 57) = 133.33, *MSE* = 0.34, *p* < .001, η_*p*_^2^ = .70. Responses to schema consistent groupings were faster than responses to schema inconsistent groupings, *F*(1, 57) = 31.59, *MSE* = 0.09, *p* < .001, η_*p*_^2^ = .36. Age and schema consistency interacted, *F*(1, 57) = 7.93, *MSE* = 0.09, *p* = .007, η_*p*_^2^ = .12, with OA showing greater slowing for schema inconsistent groupings than for schema consistent groupings. There was a main effect of cuing condition, *F*(2, 114) = 100.49, *MSE* = 0.04, *p* < .001, η_*p*_^2^ = .64, but no interaction between age and cuing condition, *F* < 1. Unlike the original ANOVA, there was an interaction between schema consistency and cuing condition, *F*(2, 114) = 4.41, *MSE* = 0.02, *p* = .014, η_*p*_^2^ = .07, which reflected somewhat stronger cuing condition effects for schema consistent groupings than for schema inconsistent groupings. Thus, the comparison between one unique cue and one unique cue plus one shared cue (memory-as-discrimination test) was significant for both schema consistent groupings, *t*(58) = 4.55, *p* < .001, and schema inconsistent groupings, *t*(58) = 2.02, *p* = .048; the comparison between one unique cue and two unique cues (encoding-retrieval match test) was significant for both schema consistent groupings, *t*(58) = 10.20, *p* < .001, and schema inconsistent groupings, *t*(58) = 7.82, *p* < .001; the comparison between one unique plus one shared cue and two unique cues (cue overload test) was significant for both schema consistent groupings, *t*(58) = 13.31, *p* < .001, and schema inconsistent groupings, *t*(58) = 7.67, *p* < .001. Finally, the triple interaction was not significant, *F*(2, 114) = 1.90, *MSE* = 0.02, *p* > .15.
